# Motivated Down-Regulation of Emotion and Compassion Collapse Revisited

**DOI:** 10.3389/fpsyg.2022.801150

**Published:** 2022-07-13

**Authors:** William Hagman, Gustav Tinghög, Stephan Dickert, Paul Slovic, Daniel Västfjäll

**Affiliations:** ^1^JEDILab, Division of Economics, Department of Management and Engineering, Linköping University, Linköping, Sweden; ^2^JEDILab, Division of Psychology, Department of Behavioral Sciences and Learning, Linköping University, Linköping, Sweden; ^3^Division for Human-Centered Systems (HCS) at the Department of Computer and Information Science, Linköping University, Linköping, Sweden; ^4^Department of Medical and Health Sciences, The National Center for Priority Setting in Health Care, Linköping University, Linköping, Sweden; ^5^Department of Psychology, University of Klagenfurt, Klagenfurt, Austria; ^6^School of Business and Management, Queen Mary University of London, London, United Kingdom; ^7^Decision Research, Eugene, OR, United States

**Keywords:** compassion, emotion, emotion regulation, down-regulation, charitable giving, prosocial behavior

## Abstract

Compassion collapse is a phenomenon where feelings and helping behavior decrease as the number of needy increases. But what are the underlying mechanisms for compassion collapse? Previous research has attempted to pit two explanations: Limitations of the feeling system vs. motivated down-regulation of emotion, against each other. In this article, we critically reexamine a previous study comparing these two accounts published in 2011 and present new data that contest motivated down-regulation of emotion as the primary explanation for compassion collapse.

## Introduction

A central question in research on charitable giving and prosocial giving is how we value human lives (Slovic, 2007). Donors are often scoped insensitive and do not increase donations as the need increases (Dickert et al., 2015; Slovic et al., 2017). Donors may even be inversely scoped sensitive—a form of “compassion collapse” or “compassion fade” has been documented, where feelings and helping behavior decrease as the number of needy increases (Kogut and Ritov, 2005a,b; Slovic, 2007; Västfjäll et al., 2014). Although this finding has been replicated in different contexts and countries (Kogut and Ritov, 2005a,b; Markowitz et al., 2013; Västfjäll et al., 2014; Kogut et al., 2015), the psychological mechanisms underlying this effect is not well understood. In an attempt to remedy this, Cameron and Payne (2011; henceforth C&P) tested an affect trigger or capacity explanation vs. a motivated emotion regulation account for compassion collapse. According to C&P, the affect trigger account suggests that emotions are more strongly elicited for individuals than groups and that as the scale increases, individuals start to lose feelings (Slovic, 2007; Genevsky et al., 2013; Västfjäll et al., 2014, 2015; Lindauer et al., 2020; Moche et al., 2020). In a sense, the affect trigger account suggests that compassion collapse occurs because of capacity limitations of the affective system and is an inherent property of compassion (and other emotions; see the extensive work on psychophysical numbing; Fetherstonhaugh et al., 1997, as well as the general decreased marginal utility in descriptive models of decision making such as prospect theory; Slovic, 2007).

In C&P’s view, the motivated emotion regulation account, on the other hand, suggests that groups indeed elicit strong emotion, but that people may engage in motivated behaviors and goal-focused emotion regulation to prevent those feelings to occur. Specifically, C&P suggested that costly helping will be avoided. They reasoned that both financial and emotional costs will be downregulated and further that these costs would be perceived as greater as the scale of helping increases. Thus, they hypothesized that compassion collapse would emerge strongly when there is a clear motivation to avoid feeling compassion for multiple victims. Previous research on compassion collapse has primarily explored individuals’ willingness to donate money toward victims (Kogut and Ritov, 2005a), and why C&P reasoned that the expectation of being asked to help may serve as a financial motivation to avoid emotions toward many victims.

As a critical test of this hypothesis, C&P conducted an experiment (Experiment 1 in C&P, 2011 published in the prestigious outlet *Journal of Personality and Social Psychology*) where the motivation to regulate emotion was experimentally manipulated. In this study, 120 participants first read about one or eight children in Darfur (a common between-subjects manipulation in compassion collapse studies; Kogut and Ritov, 2005a). Second, C&P introduced the critical between-subject manipulation where participants either (a) rate their feelings toward the children or (b) first rate their feelings toward the children *and* then respond to how much money they would be willing to donate. We will refer to “a” as the no-help request condition and “b” as the help request condition. Critical to the current article, the no-help request condition (a) did not explicitly state that subjects would not be asked to make a donation decision. Since C&P argued that for the no-help request condition, they did not explicitly state that the participants would not be asked to donate because *such instructions* could have inadvertently focused participants on the idea of donating. Following this manipulation, participants in both conditions rated their feelings on a 9-item compassion scale that included statements such as “how sympathetic do you feel toward the child (children)?” and “how compassionate do you feel toward the child (children)?”

C&P predicted and found that there was no main effect on help request (donation-no donation) or the number of children (1 vs. 8) on rated compassion, but a significant interaction where the compassion collapse (greater compassion for one over eight) only occurred in the donation condition. In the no-donation condition, the pattern reversed so that compassion was instead greater for the eight children.

C&P interprets these findings as suggesting that the driving mechanism behind compassion collapse is active down-regulation of emotion that only occurs *when people themselves expected to help*—a finding that supports the motivated emotion regulation account over the affect trigger explanation. While this is only studying one of three studies in the C&P 2011 paper, this particular finding has been cited several times in support of the notion of “empathy/compassion as a choice” (Zaki, 2014; Cameron, 2017; Cameron et al., 2019, 2022; Scheffer et al., 2021) and is an important contribution for a critical experiment demonstrating a boundary condition for compassion collapse.

In this article, we revisit this experiment and present new data that suggest that the experimental manipulation of motivated emotion regulation used by C&P is problematic and consequently is limited as an explanation for compassion collapse. We identified several critical methodological problems in the original study and we present new data from a large-powered study (using the original materials) that experimentally manipulates or measures the methodological concerns identified with the original study.

## Problems With C&P 2011 Experiment 1

1. First, even though the compassion collapse effect has been replicated across more than 20 studies (as cited in the C&P paper), the published effects range from medium effect sizes in the expected direction (Kogut and Ritov, 2005a,b; Moche et al., 2022) over null effects (Dickert et al., 2016; Hart et al., 2018) to small effects in the opposite direction (more giving to the many: Wiss et al., 2015). Thus, a minor, but still noteworthy aspect of Experiment 1 in C&P (2011) is the relatively small sample size (roughly 30 participants per condition: a total of 120). This *n* is just on the border of the minimal sample adequate to detect medium effect size in an interaction (*f* = 0.25; Cohen, 1988; *n* = 128 required at 0.80 power), but would not be able to detect a small effect (*f* = 0.10), where an *n* = 787 would be required (computed using G*Power 3.1).

2. Second, and much more critical, is the item in the main dependent variable—the compassion scale. While most of the items are standard sympathy and distress items, one item (henceforth called the “Give money” item) of the scale is formulated: “*To what extent do you feel that it is appropriate to* ***give money*** *to aid the child (children)?”* (Emphasis added by current authors). Given that this item was included in the main dependent variable that was used in both the help request and no-help request conditions, it could be argued that the inclusion of this item may effectively wipe out any expected difference between conditions. Participants in the no-help request condition would arguably come to expect that they would be asked to donate. This is particularly interesting, as C&P told participants in no-donation condition explicitly that they would not be asked to donate *“because such an instruction might have seemed like a violation of conversational norms and might have inadvertently focused participants on the idea of a donation even as we assured them of its absence.*” (p. 4). We argue that it is an equally big risk to focus participants on donation by including that very question as an item in the scale, and further, to use this scale as the main dependent variable. This methodological problem casts doubt on the effectiveness of the donation/no-donation manipulation, even though the original study found a condition difference in the expected direction.

Arguably, if donation requests have an effect, then the order of the donation item in the compassion scale could make a difference so that if appearing first, it would have a larger influence than if appearing last. We contacted C&P (personal communication) to clarify if there were any order effects and learned that the order was fixed so that the Give money item was always randomized to occur in order 5, 6, 7, or 8 position of the 9-item scale. In the current experiment, we systematically manipulate the serial position of the give money item in the compassion scale so that either the Give money item question appears first or last. If the Give money item indeed cues thinking on donations, we should see a larger effect when it appears first than when it appears last.

3. Third is related to the second concern and was also raised by C&P: Even when not asked for a donation, participants may have expected a donation request because the materials depicting victims are often based on actual charity advertisements with the purpose to solicit donations. Given that C&P modeled their stimuli on those in studies conducted by Kogut and Ritov (2005a,b) and that the information given about the victims (west Darfur civil war victims suffering from deadly diseases such as malaria, dysentery, and cholera) is very typical of charity requests (Erlandsson et al., 2016, 2018), it is likely that even participants in the no-donation condition may have been cued to think about donations. Simply asking participants to state their expectations about if they would be asked to donate or if they thought about donating during the stimuli presentation would have given information about this, but C&P does not present any such data. We believe that participants in both conditions may have thought about donating at some point and an important piece of information that is missing is, if so, what was the prevalence, and was it different between conditions? Perhaps fewer participants in the no-donation condition thought about donating and thus the manipulation was (relatively) successful. On the other hand, if there was a roughly equal proportion of participants in both conditions that report thinking about donating, the effectiveness of the manipulation must be seriously questioned. We cannot give estimates of the prevalence of thoughts about donating with the existing data from the C&P article, and together with the problems identified with the compassion scale, this is a central point to evaluate the validity of the manipulation and findings. In the present article, we therefore also measured people’s self-reported expectations about donating as well as thoughts about donating in both the help request and no-help request conditions to assess if the manipulation worked as intended.

4. Fourth, given the potential problem that the Give money item in the compassion scale resembles the help request manipulation, it is problematic that it is used as the only dependent variable to measure compassion collapse as C&P did. In the present article, we include measures of donation (both a yes-no decision as well as amount; a standard way of probing helping intentions: Dickert et al., 2011) in both conditions (but presented last in the session and without participant’s prior knowledge). Furthermore, given that a central feature of C&P’s account for explaining compassion collapse is emotion regulation, we measure self-reported mood at several points (baseline, after picture, and after donation) to directly estimate the hedonic consequences of any emotion regulation attempts. Thus, we can independently, and using a measure that is not potentially contaminated by eliciting thoughts about donation, assess if emotion regulation is more effective in the donation than in the no-donation conditions as predicted by C&P.

We conducted a high-powered replication (sample size sufficient to detect a small effect) of Experiment 1 from C&P (2011) with additional measures and manipulations to help clarify the issues stated above. As C&P argued, evidence of the motivated emotion regulation account would be found if compassion collapse (more giving to the one than the eight) occurred only in the help request conditions but not in the no-help request condition. Assuming that the between-subjects manipulation would replicate and taking into account our concerns with the main dependent variable, we expected a three-way interaction so that the compassion collapse effect should be stronger when the critical Give money item is presented first (as opposed to last) in the help request condition and induce compassion collapse in the no help request condition. Further, the motivated emotion regulation account suggests that our additional measures (donation and repeated assessment of mood) should show similar effects (e.g., less giving and better mood in the no-donation conditions). Thus, we seek to test this prediction to see if additional support for the emotion regulation account can be obtained. Finally, we assessed the prevalence of thoughts of donating and expectations to donate in all conditions. If the manipulation of expecting to donate is successful, significantly fewer participants should report thinking about donating or expecting to donate in the no-donation conditions.

## Materials and Methods

### Design

A total of 1,177 participants (53.1% female, ages 18–82 years, mean 39.55) were recruited from a US sample by the Decision Research, Eugene, OR, to complete this study online.

We followed the 2 × 2 design used by C&P so that participants were randomly assigned to read about one or eight children from Darfur (number of victims) or was given the expectation that they would have to report a donation amount later in the experiment or that they would just be asked to rate their emotions toward the child (children) (help request). The sample sizes in each of these four cells ranged from a minimum of 290 to a maximum of 307.

In addition, a novel design feature was introduced to examine the potential attenuating effect of the Give money item (*To what extent do you feel that it is appropriate to* ***give money*** *to aid the child (children)* on the help request manipulation. Roughly half of the participants in each group received either the Give money item in the compassion scale as the first or the last item in their rating of compassion. The resulting design was a 2 (number of victims) × 2 (help request) × 2 (Give money item placement) between-subjects design (with a range of 135–155 participants per cell). The critical dependent variable was self-reported compassion toward the child (children) measured with the same compassion scale used by C&P.

### Procedure

All critical stimuli and instructions are identical to C&P. We included an additional mood measure so that after viewing an introductory page, the participants answered demographic questions and the mood rating question “Overall how do you feel right now?” on a Likert scale ranging from –10 (Very Negative) to 10 (Very Positive). Participants then saw the same information about Darfur as presented in C&P where they either saw one or eight child images (with names and ages), depending upon the victim condition. Like the original study, the images and text were displayed on the screen for 1 min.

Participants were then given the donation manipulation. In the *help request condition*, they were told the following: “Later in the experiment, you will be asked to rate your emotions toward this child [these children] and report how much money you would be willing to donate.” Before viewing the images, participants were reminded, “Remember that later in the experiment, you will be asked to rate how you feel toward the child [children] you saw and how much you would be willing to donate.” In the *no-help request condition*, participants were told the following: “Later in the experiment, you will be asked to rate your emotions toward this child [these children]. Remember that later in the experiment, you will be asked to rate how you feel toward the child [children] you saw.” Participants in both conditions then saw the same Darfur information and images for min. They completed the 9-item scale from C&P measuring compassion-related feelings and attitudes toward the target or targets of aid and also answered the mood-rating question “Overall how do you feel right now?” on a Likert scale ranging from –10 (Very Negative) to 10 (Very Positive). To be as close as possible to the original study, we included the same series of scales measuring alternative explanations for the collapse of compassion as used by C&P (in all conditions). These measures did, however, not yield any additional information and are therefore not presented here. Following this, we asked participants to rate their thoughts about donating as well as their expectations about being asked to donate (not included in the original C&P study).

### Thoughts of Donation and Expecting to Donate

Participants were asked “When you were rating your emotions toward this child (children), did you reflect on whether or n not to donate anything to the child (children)? (reflect item)” and “Did you expect that you were going to be asked to donate money to the child (children)” (expect item). Both measures had the answer alternatives “yes” or “no.”

Next, participants responded to a hypothetical donation question (Dickert et al., 2011) that first contained the ‘‘yes’’ or ‘‘no’’ question: ‘‘Imagine you had $25 dollars in your wallet right now. Would you be willing to donate money to help the children shown earlier in the survey?’’ Participants that answered ‘‘yes’’ could indicate with a slider the amount they would donate (0-25 dollars). Finally, participants answered the mood-rating question again followed by a short version of the difficulties in emotion regulation scale^1^ (DERS).

## Results

**FIGURE 1:**
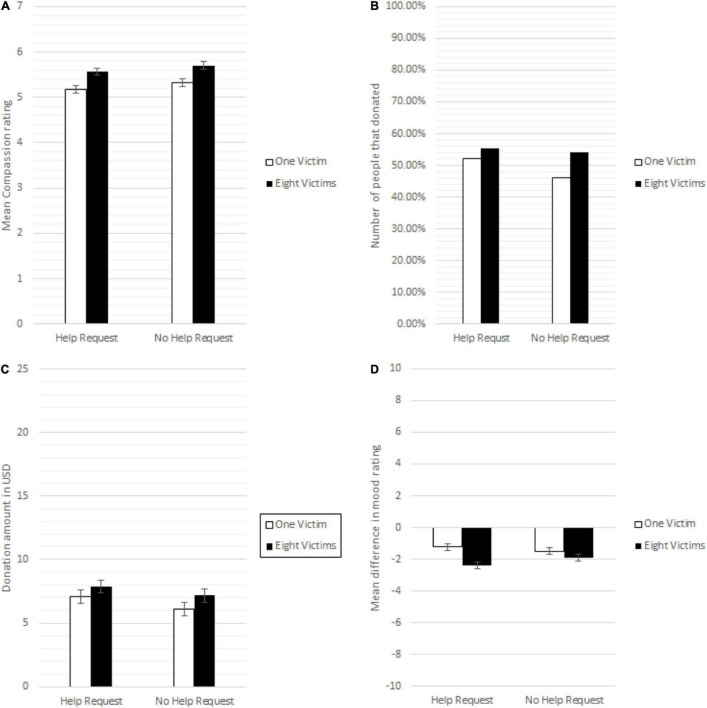
**(A)** Mean compassion toward the child (children) across conditions (higher number indicates more compassion). **(B)** Number of participants that choose to donate across conditions. **(C)** Mean donation amount in USD split by help request and number of victims’ condition. **(D)** Mean change in mood ratings between baseline and after viewing the child/children (lower number indicates a more negative mood). Error bars are SD.

Following the analysis strategy of C&P, we averaged the nine items in the compassion scale (Cronbachs α = 0.96) and two-way between-subject analysis of variance (ANOVA) was conducted to examine the effect of the number of victims and help requests on compassion. As shown in Figure 1A and contrast to the C&P’s results, we find a significant main effect of the number of victims on rated compassion (i.e., participants felt more compassion for the many), *F*(1, 1,173) = 23.549, *p* = 0.000, η*_*p*_^2^* = 0.020. Compassion was higher for the eight than for the one child, both in the help request and no-help request conditions. There was no significant effect of help request on rated compassion, *F*(1, 1,173) = 3.328, *p* = 0.068, η*_*p*_*^2^ = 0.003. Importantly, the interaction term between help request and the number of victims, which was used as the critical outcome in C&P, was not significant, *F*(1, 1,173) = 0.012, *p* = 0.913, η*_*p*_*^2^ = 0.000.

To further test if the interaction between help requests and the number of victims could be detected with other outcome measures, we first conducted an analysis of the donation data. For the binary donation decision, 613 out of the 1,177 participants chose to donate. When split by conditions, the donation patterns resemble the compassion ratings, where slightly more participants donated when presented with eight children (in both the help request conditions). However, this effect failed to reach significance difference, *X*^2^(1, 613) = 0.890, *p* = 0.372 (see Figure 1B).

A two-way between-subject analysis of variance (ANOVA) was conducted to examine the effect of number of victims and help request on donation amount (including zero). Here, neither the main effect of number of victims, *F*(1, 1,173) = 3,335, *p* = 0.068, η*p*^2^ = 0.003, nor the main effect for help request *F*(1, 1,173) = 2,722, *p* = 0.099, η*p*^2^ = 0.002, were significant. Similar to the results for the compassion scale, the expected interaction between help request and number of victims was not significant, *F*(1, 1,173) = 0.099, *p* = 0.754, η*_*p*_*^2^ = 0.000 (Figure 1C).

Next, we examined the mood ratings (pre-post difference) with a two-way between-subject analysis of variance (ANOVA). As shown in Figure 1D, we find a significant main effect of number of victims on difference in mood (i.e., participants felt worse after seeing many children), *F*(1, 1,173) = 15.473, *p* = 0.000, η*_*p*_*^2^ = 0.013. There was no significant effect of help request on difference in mood, *F*(1, 1,173) = 0.297, *p* = 0.856, η*_*p*_*^2^ = 0.000. Moreover, the interaction term between help request and number of victims was not significant, *F*(1, 1,173) = 3.194, *p* = 0.074, η*_*p*_*^2^ = 0.003.

Taken together, the compassion scale, donation data, and mood change ratings failed to show the expected interaction between the number of victims and help requests. The main dependent variable from C&P, the compassion scale, showed that participants experienced higher levels of compassion for eight (over one) children in both the help and no-help request conditions. This finding is consistent with some previous research on compassion collapse using different materials and contexts (Wiss et al., 2015) but was not expected since we used the same materials as C&P. Thus, we fail to directly replicate the effect of Experiment 1 in C&P.

### The Potential Problem With the Compassion Scale

**FIGURE 2:**
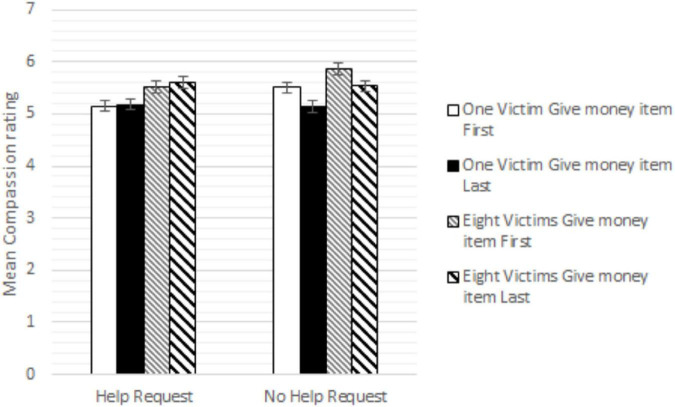
Mean compassion toward the child (children) across conditions. Error bars are SD.

The failure to replicate the compassion collapse effect may be related to the issue of the serial position of the Give money item [To what extent do you feel that it is appropriate to give money to aid the child [children)] in the compassion scale as outlined above. We thus conducted a 2(number of children) × 2(help request) × 2 (placement of Give money item; first/last) ANOVAs on the compassion ratings. As shown in Figure 2, there was no significant effect of order of the Give money item, *F*(1, 1,169) = 3.231, *p* = 0.073, η*_*p*_*^2^ = 0.003. Further, there was no interaction between the number of victims and order of the Give money item, *F*(1, 1,169) = 0.095, *p* = 0.758, η*_*p*_*^2^ = 0.000. However, there was a significant interaction between the order of the Give money item and help request, *F*(1, 1,169) = 6.527, *p* = 0.011, η*_*p*_*^2^ = 0.006, implying that, if anything, the participants rated higher compassion when prompted early with the “giving money” item. The three-way interaction between number of victims, help request, and the placement of the Give money item did not reach significance; *F*(1, 1,169) = 0.016, *p* = 0.900, η*_*p*_*^2^ = 0.000.

We conclude that the serial position of the Give money item did not have a substantial effect on the compassion ratings measured with the original scale used by C&P. *A priori*, we argued that the inclusion of the item could have minimized the effect of the critical help request manipulation and that if placed earlier, it may have had a larger attenuating effect (compared to if it occurred last). We do not find support for this but given that we also could not replicate the critical two-way interaction between the number of children and help requests found in C&P, it is inconclusive what role the serial position of this item played in the original C&P study.

A related concern about the help request manipulation was that participants in both conditions may be either thinking about donations (because of the nature of the stimulus material which is very similar to charitable ads; Erlandsson et al., 2016) or expecting to donate (because showing suffering child victims typically is associated with help requests; Erlandsson et al., 2016).

In the help request conditions, which explicitly stated that participants would: “Report how much money you would be willing to donate,” 33.4% of the participant **did not** expect to be asked to donate money (measured using the “expect item”), suggesting that even explicitly instructing participants is not a guarantee that they will believe that they will be asked to donate. More central to the manipulation and the interpretation of our failure to replicate the original findings, in the no-help requested condition, only 41.3% reported that they **did not** expect that they would be asked to donate. Furthermore, a majority of the participants in both the help requested (53.4%) and no-help requested (59.2%) conditions stated that they did think about donating to the child (children) when rating their emotions (as measured by the “reflect item”). Combined, these results cast serious doubt on the validity of the manipulation and may partly account for the fact that we could not replicate C&P findings.

**FIGURE 3:**
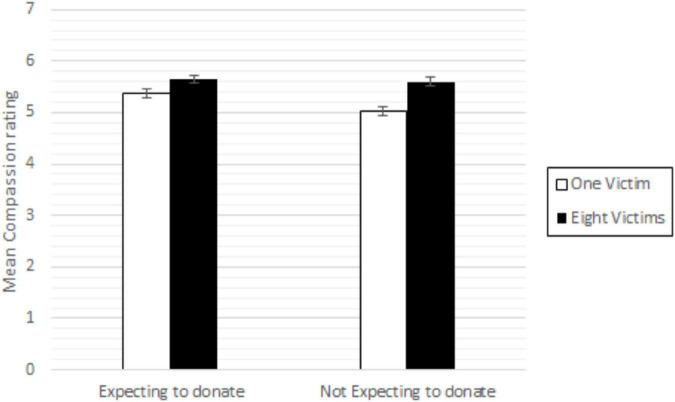
Mean compassion toward the child (children) across conditions (higher number indicates more compassion). Error bars are SD.

The “expect” and “reflect” items provide another quasi-experimental approach for studying compassion collapse. A central prediction from C&P is that compassion collapse should occur for those that expect to donate. Thus, we used the expect and reflect items as categorical variables substituting the help request variable. A two-way between-subjects ANOVA was conducted to examine the effect of expecting to be asked to donate (expect/not expect) and the number of victims on compassion. A significant main effect was found for number of victims, *F*(1, 1,173) = 26,679, *p* = 0.000, η*_*p*_*^2^ = 0.022, where again compassion was higher for the many, while the main effect of expecting to donate, *F*(1, 1,173) = 5,631, *p* = 0.018, η*_*p*_*^2^ = 0.005, was significant, but with higher compassion for the participants expecting to donate (Figure 3). A two-way ANOVA of the reflect item (think/did not think) and the number of victims was conducted on the compassion scale, where a similar pattern emerged: A significant main effect for the number of victims, *F*(1, 1,173) = 23,438, *p* = 0.000, η*_*p*_*^2^ = 0.020, was found where, again, compassion was higher for the many. Further, a main effect of reflecting, *F*(1, 1,173) = 95,160, *p* = 0.000, η*_*p*_*^2^ = 0.075, was found, with higher compassion for the participants reflecting on donation. All in all, even using these items, we again fail to replicate the expected two-way interaction from C&P.

Given our earlier concerns about the validity of the manipulation, this analysis allowed us to capitalize on participants’ reports to categorize the entire sample, independent of the experimental help request manipulation, as expecting vs. not expecting to donate. Using this quasi-experimental approach, which arguably should have maximized the possibility to replicate the original number of victims × help request interaction, we still fail to find an effect.

## General Discussion and Conclusion

Compassion collapse is a central concept in the psychology of giving and has received much attention in the literature (Kogut and Ritov, 2005a; Slovic, 2007; Västfjäll et al., 2014, 2015; Erlandsson et al., 2016; Västfjäll and Slovic, 2020). What is lacking still is a comprehensive account of the mechanisms underlying compassion collapse. The motivated emotion regulation account suggested by C&P (2011) was a much-needed attempt to shed further light on the underlying driving forces and boundary conditions of this effect. While we feel that the original account of compassion collapse (the by C&P so-called affect trigger account: Slovic, 2007), suggesting that our affect system has limitations in dealing with large numbers, is in no way incompatible with the emotion regulation account suggested by C&P, we do see great merit in the notion of anticipated financial and emotional cost as a motivator of feelings and behavior. In fact, in previous work, it has been suggested that “psychic numbing”—the automatic or motivated down-regulation of emotion—is one of the key mechanisms behind emotion collapse (Slovic, 2007; Slovic and Västfjäll, 2010; Dickert et al., 2012, 2015). We do, however, disagree with the view that anticipated financial and emotional cost is the **single** driver of compassion collapse. More specifically we take issue with how Experiment 1 of C&P can be taken as evidence for motivated down-regulation. *A priori*, our main concerns with this study by C&P centered around four key issues: (1) The relative small n in the original study, (2) the inclusion of a donation item in the main dependent variable, (3) the possibility that the critical help request manipulation was ineffective, and (4) the sole reliance on a single dependent variable that may attenuate the difference between conditions. Of these four original concerns, three main issues deserve special attention.

### The Compassion Scale

The main dependent variable in C&P’s experiment 1 contained an item asking for donations—although the critical manipulation of help request was explicitly instructing participants that they would be asked to donate vs. explicitly only mention that participants would rate their feelings. We argue that the inclusion of this item might effectively erase or minimize the expected effect of the help request condition. Thus, in the present study, we systematically varied the serial position of the give money question (first vs. last) based on the prediction that if the item occurred early, it would more strongly attenuate the difference than if it did occur last. We did not find this pattern. Instead, participants rated higher compassion when prompted with giving money in the no-help request condition.

### Failure to Replicate the Critical Interaction

Most central to the motivated emotion regulation account, we were unable not find any evidence across four measures (the original compassion scale used by C&P, donation decision, donation amount, and mood ratings) of the critical number of children × help request interaction. C&P’s main finding was that compassion collapse (giving more to 1 over 8 children) only occurred in the donation request condition and not in the no-request condition—a finding that is interpreted as evidence of motivated down-regulation of emotion when the anticipated cost is high (i.e., when participants expected to donate). Given that we were not able to find this, even though we used a large sample enough to detect a small effect sheds some doubt on the validity of the original finding. Admittingly, even though we used the same materials and presentation of stimuli as the original study, there were some differences between our study and C&P (2011): (a) C&P used a student sample, whereas we used two more heterogeneous and representative samples and (b) participants in the original C&P study was tested individually, whereas our samples responded online. We believe the sample issue to be a relatively small difference, whereas it is possible that the procedural difference (laboratory vs. online) between our studies may have played a role. For instance, participants in our study may have not engaged emotionally in the same way as participants in the original study. However, it appears from both the compassion ratings and the mood measure that our manipulations emotionally affected the participants. Importantly, other research shows that compassion collapse can be observed using online samples (Moche et al., 2022), and research in other related domains of decision-making has shown that results from online samples are very similar to those obtained in the lab (Birnbaum, 2000; Ruggeri et al., 2020). Thus, it is unlikely that differences in sample and procedure between the studies would account for the differences in results. If motivated down-regulation occurs only in a tightly controlled laboratory setting, then this would be a severe limitation of this account of compassion collapse.

### The Help Request Manipulation

Drawing on the reasoning used by C&P themselves (see section “Introduction”), we argue that it is very likely that participants in both the help request and no-help request conditions spontaneously thought about donation and maybe even have expected to donate. This is especially likely since the conversational norm activated by showing starving African child victims combined with a story about need most likely is an expectation to be asked to help. However, this may be less of a problem if the proportion of participants thinking about and expecting to donate is substantially lower in the no-help request condition than in the help request condition. When we asked participants to what extent they thought about donations, more than 50% in both conditions reported “yes” with no significant difference between conditions. Even more critical is that when asked if they expected to be asked to donate, over 50% of participants in the no-help request answered “yes” and less than 70% in the help request (that were explicitly instructed that they would be asked to donate) answered “yes.” This finding is unlikely solely driven by the failure of comprehension since recent studies have shown that online samples typically perform much better on comprehension tests of instruction typically used in psychology studies than do undergraduate samples (Hauser and Schwarz, 2016). Instead, this finding likely reflects the fact that when showing experimental stimuli resembling what participants typically see in charitable ads, they spontaneously think about how to help and thus expect to be asked this question at some point during the experiment. These findings undermine the validity of the help request manipulation but also presented a possibility for us to perform another test of the motivated emotion regulation account. Following the same logic as used by C&P, we reasoned that participants reporting that they expected to donate would show compassion collapse, whereas participants not expecting to donate would not show this effect. Thus, we substituted the help request variable (i.e., an intention to treat analysis) with the quasi-experimental variable self-reported expectation (expected vs. did not expect; a per protocol analysis). This analysis should have maximized the possibility to find the expected interaction, but here the effect was significant in the opposite direction, namely that people that expected to donate gave higher ratings of compassion. Taken together, these findings suggest both that help request manipulation may not be very effective and that even when relying on participants’ reports about expecting to donate, it is difficult to find evidence for the motivated emotion regulation account.

In summary, this high-powered replication of Experiment 1 in C&P (2011) failed across multiple measures (including controlling for a potentially confounding item in the original main dependent variable) and ways of categorizing/testing the help request manipulation. These results naturally do not invalidate the entire emotion regulation account of compassion collapse, but they suggest that the often-cited findings from experiment 1 of C&P may be difficult to replicate. Therefore, the implications of this particular study should be interpreted with caution. Recent work by Cameron et al. (2019, 2022) on the role of empathy and compassion (Scheffer et al., 2021) as a choice relies heavily on the results of experiment 1; for example, Cameron (2017) argues that compassion collapse should **only** emerge when people are motivated to avoid compassion for multiple victims and when they engage in emotion regulation processes to reduce compassion for multiple victims. In pitting the two views against each other, Cameron (2017) further argues that manipulating motivation and emotion regulation should not influence compassion collapse according to the “capacity” account. The evidence of the motivational account is then summarized with an explicit focus on study 1 in C&P and Cameron (2017) later concludes: “*One take-home message is that change is possible: unlike the claims of capacity accounts, the motivational account suggests that people can choose to feel more compassion for mass suffering*” (p. 265).

While it is possible that compassion may be subject to active choice, the results of the current study suggest that role of active down-regulation of compassion when expecting financial and emotional costs are salient is still open for interpretation and discussion. Given the current results, it appears difficult to fully refute the affect trigger/capacity account of compassion and empathy based on the original C&P Experiment 1 alone. Therefore, a continued active research program and discussion on the boundary limits and driving forces behind compassion collapse are much needed.

## Data Availability Statement

The raw data supporting the conclusions of this article will be made available by the authors, without undue reservation.

## Ethics Statement

The studies involving human participants were reviewed and approved by the Decision Research IRB. The patients/participants provided their written informed consent to participate in this study.

## Author Contributions

WH collected data and data analysis. DV contributed to project management and drafted manuscript. GT, SD, and PS revised writing. All authors contributed to the article and approved the submitted version.

## Conflict of Interest

The authors declare that the research was conducted in the absence of any commercial or financial relationships that could be construed as a potential conflict of interest.

## Publisher’s Note

All claims expressed in this article are solely those of the authors and do not necessarily represent those of their affiliated organizations, or those of the publisher, the editors and the reviewers. Any product that may be evaluated in this article, or claim that may be made by its manufacturer, is not guaranteed or endorsed by the publisher.

## Footnotes

1 DERS had a significant main effect F(1, 436) = 15.223, p = 0.000, ηp2 = 0.034, where participants with lower score on DERS felt more compassion compared to participants with higher scores, but no significant interaction effects for the critical comparison number of victims F(1, 440) = 0.476, p = 0.491 ηp2 = 0.001 or with help request F(1, 440) =,794, p = 0.373, ηp2 =0.002.
